# Synthesis, characterization and surface properties of an organosilicon surfactant

**DOI:** 10.3389/fchem.2026.1744604

**Published:** 2026-01-30

**Authors:** Xinhua Zhu, Yanyu Wang, He Huang, Yuqiang Zhang, Xuhong Jia, Maoyong Zhi

**Affiliations:** 1 College of Civil Aviation Safety Engineering, Civil Aviation Flight University of China, Guanghan, Sichuan, China; 2 Civil Aircraft Fire Science and Safety Engineering Key Laboratory of Sichuan Province, Civil Aviation Flight University of China, Guanghan, Sichuan, China

**Keywords:** aggregation behavior, organosilicon surfactant, surface activity, thermal stability, thermodynamic analysis

## Abstract

In order to explore alternative substances for traditional fluorocarbon surfactants while maintaining high surface activity, this study successfully designed and synthesized an organosilicon surfactant (SiCH) containing a hydroxyl quaternary ammonium salt. Its molecular structure was confirmed by Fourier transform infrared spectroscopy (FT-IR), nuclear magnetic resonance (NMR), and mass spectrometry (MS). This surfactant exhibits excellent thermal stability, with an initial decomposition temperature of 167 °C and minimal residue at high temperatures. The critical micelle concentration (CMC) and the surface tension (*γ*_CMC_) at the CMC of SiCH are 9.10 mmol/L and 22.81 mN/m, respectively. The organosilicon surfactant–sodium hexanesulfonate (SiCH-SHS) exhibits significant synergistic effect, reducing the CMC and *γ*_CMC_ to 6.88 mmol/L and 21.03 mN/m respectively. The area occupied by a single surfactant molecule at the air/water interface (*A*_S_) of the SiCH-SHS is 1.24 nm^2^, and the pC_20_ is 2.91. The size distribution of SiCH is unimodal, and they can self-assemble into non-uniform sized spheroidal aggregates (122–295 nm). Moreover, the thermodynamic process of micelle formation of the surfactant was studied through conductivity tests conducted at different temperatures (298.15 − 303.15 K). Thermodynamic analysis indicates that micellization is a spontaneous ( ${\Delta G}_{m}^{0}$ <0), entropy-driven process.

## 1 Introduction

Surfactants are a class of amphiphilic compounds characterized by their ability to adsorb at interfaces and significantly reduce surface or interfacial tension. Due to their unique molecular structure, consisting of a hydrophilic head and a hydrophobic tail, they exhibit distinct properties such as wetting, emulsification, and foaming (Cheng et al., 2022). Consequently, simple surfactants are extensively used in various industrial sectors, including detergency, oil recovery, and fire extinguishing formulations (Zhao et al., 2024).

Fluorocarbon surfactants exhibit distinct surface activity, excellent thermal stability and chemical stability, and are widely used in industry (Lu et al., 2010; Sha et al., 2015; Zaggia and Ameduri, 2012). They can reduce the surface tension of water to below 20 mN/m, which is significantly superior to traditional hydrocarbon surfactants (30–40 mN/m) (Wang et al., 2017; Zhu et al., 2020). However, fluorocarbon surfactants—specifically per- and polyfluoroalkyl substances (PFAS) such as perfluorooctanesulfo-nic acid (PFOS) and perfluorooctanoic acid (PFOA)—exhibit high environmental persistence owing to their chemically stable molecular structures. These compounds accumulate in aquatic and terrestrial systems and can bioaccumulate through the food chain (Malik et al., 2024). In response, global regulatory measures have been progressively implemented, such as the restriction of PFOS under the Stockholm Convention, accelerating the development of short-chain fluorinated and fluorine-free alternatives (Joshi et al., 2025). Obviously, the development of fluorine-free surfactants is the fundamental strategy to solve the problem of fluorine pollution.

As is well known, silicone surfactants are a new class of ones with excellent surface activity that consist of a unique siloxane backbone, which can significantly reduce the surface tension of water to approximately 20 mN/m (Snipes et al., 2023; Xiao and Zhao, 2018). The siloxane chains in the structure of silicone surfactants are flexible, which causes the methyl groups to be arranged more closely at the air/water interface. The results endow them with superior surface activity in both aqueous and nonaqueous solutions (Svitova et al., 1996). Compared with conventional fluorocarbon-based surfactants, silicone variants are free of persistent pollutants, demonstrate higher biodegradability, and pose considerably lower long-term ecological risks to soil and aquatic systems. These attributes position silicone surfactants as promising candidates for achieving high surface activity together with improved environmental safety (Zhao et al., 2024). Therefore, more and more scholars are dedicated to the synthesis of high surface activity organosilicon surfactants and their application as alternatives to fluorine-based surfactants. Liu et al. prepared a series of carbosilane quaternary ammonium surfactants and found that the surface tension could be reduced to 30.5 mN/m at a concentration of 0.14 mmol/L (Liu et al., 2022). Du et al. studied the aggregation behavior of two trisiloxane-tailed surfactants in aqueous solution (Du et al., 2014). The results show that the critical micelle concentration (CMC) was 15.5 mmol/L, with the surface tension (γ_CMC_) at the CMC as low as 23.0 mN/m. The surface activity of these silicone surfactants was somewhat superior to that of conventional surfactants, and may improve their potential applications in colloids and interfaces science. Etz et al. investigated the high-temperature decomposition chemistry of trimethylsiloxane surfactants based on quantum mechanical methods (Etz et al., 2022). The analysis indicated that trimethylsiloxane surfactants are potential substitutes for PFAS in fire suppression technologies. Fang et al. investigated the aggregation behavior of two cationic silicone surfactants in aqueous solution, and found that the γ_CMC_ of the two surfactants can be reduced to 20 mN/m (Fang et al., 2017). Moreover, it was discovered that these surfactants had excellent extraction ability for several metal ions in chloroform.

In this work, an organosilicon surfactant with excellent surface activity is proposed to be synthesized via quaternization using 3-chloropropyltrichlorosilane and N,N-dimethylisopropanolamine as raw materials. The obtained surfactant is systematically evaluated in terms of thermal stability, surface activity, micellar thermodynamic properties and foam performance. Furthermore, from the perspectives of cost and efficiency, in practical applications, multiple surfactants are often used in combination. Therefore, the compatibility performance of organic silicon and hydrocarbon surfactants was further evaluated. It provides a theoretical basis for the replacement of fluorine-based surfactants and their application in the fields of colloid and interface science.

## 2 Materials and methods

### 2.1 Materials

3-Chloropropyltrichlorosilane (97%), chlorotrimethylsilane (98%), N,N-dimethylisopropanolamine (98%), and isopropyl alcohol (99%) were purchased from Shanghai Aladdin Biochemical Technology Co., Ltd. Methanol (99.5%), n-hexane (97%) were supplied by Chengdu Jinshan Chemical Reagent Co., Ltd. Sodium 1-hexanesulfonate (98%) was obtained from Shanghai Macklin Biochemical Co., Ltd., and deionized water was provided by Chengdu Kelong Chemical Co., Ltd.

### 2.2 Synthesis of surfactant

Organosilicon surfactants were synthesized by a two-step process. The first step involved the preparation of a silicone-based intermediate through sequential alcoholysis and hydrolysis reactions, followed by a quaternization step to introduce the functional groups and yield the final product.

Step 1: Synthesis of intermediate [(CH_3_)_3_SiO]_3_SiCH_2_CH_2_CH_2_Cl

**FIGURE 1:**
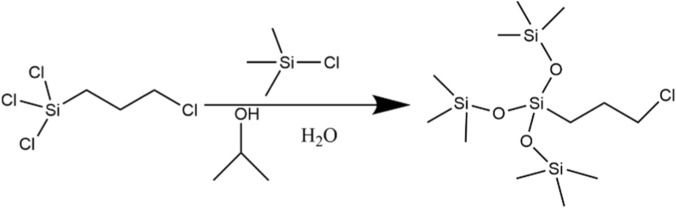
Synthesis of intermediate [(CH_3_)_3_SiO]_3_SiCH_2_CH_2_CH_2_Cl.

A four-neck flask, equipped with a mechanical stirrer, thermometer, constant-pressure dropping funnel, and reflux condenser, was filled with 3-chloropropyltrichlorosilane (0.05 mol, 10.93 g) and trimethylchlorosilane (0.20 mol, 22.17 g). The mixture was stirred at 25 °C, then the isopropanol (0.45 mol, 27.32 g) was added slowly to initiate the alcoholysis reaction. Subsequently, deionized water (0.45 mol, 8.12 g) was introduced dropwise into the flask. The mixed solution was further stirred at 25 °C for 3 h. After completion, the organic layer was separated, washed repeatedly with deionized water until neutral pH was achieved, and dried over molecular sieves overnight. The solvent was removed by rotary evaporation, and the product was purified by reduced-pressure distillation to afford chloropropyltris (trimethylsiloxy)silane, corresponding to a yield of 93%. The synthesis pathway is illustrated in Figure 1.

Step 2: Synthesis of target products ([(CH_3_)_3_SiO]_3_Si(CH_2_)_3_^+^N(CH_3_)_2_CH_2_CHOHCH_3_)·Cl^−^

**FIGURE 2:**
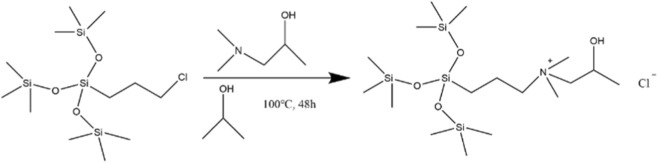
Synthesis of surfactant ([(CH_3_)_3_SiO]_3_Si(CH_2_)_3_^+^N(CH_3_)_2_CH_2_CHOHCH_3_)·Cl^−^.

The synthesized intermediate (0.02 mol, 7.46 g), N,N-dimethylisopropanolamine (0.03 mol, 3.10 g) and isopropanol solvent (16 mL) were placed in a three-neck flask equipped with a reflux condenser. The mixture was stirred and heated to 100 °C in an oil bath under reflux for 48 h. After completion, excess N,N-dimethylisopropanolamine and the solvent were removed by reduced-pressure distillation. The target product (SiCH) was obtained by vacuum distillation, wash with anhydrous methanol and n-hexane (1:5 volume ratio), and vacuum drying, corresponding to a yield of 89%. The reaction scheme is presented in Figure 2.

### 2.3 Characterization

The molecular structure of the synthesized organosilicon surfactant was characterized using Fourier transform infrared spectroscopy (FT-IR, Thermo Scientific Nicolet iS50), nuclear magnetic resonance spectroscopy (^1^H and ^13^C NMR, Bruker Avance III HD, 600 MHz), and mass spectrometry (MS, Waters ZQ2000). Thermal stability was assessed by thermogravimetric analysis (TG), and derivative thermogravimetry (DTG). NMR spectra were obtained with an internal standard and chloroform-*d* (CDCl_3_) as solvent. IR spectra were performed on the surfactants dispersed in anhydrous KBr pellets.

The surface tension of the single system (the organosilicon surfactant, SiCH) and the mixed system (the organosilicon surfactant–sodium hexanesulfonate, SiCH-SHS) were measured by the Wilhelmy plate methos using a Kino A601 tensiometer (Boston, MA, United States). All measurements were conducted at 25.0 °C± 0.1 °C. The surfactant solutions were prepared in a concentration range of 50 to 0.001 mmol/L. Before the test, the Wilhelmy plate of the surface tension meter need to be cleaned thoroughly and then burned with the flame of an alcohol lamp before immersing it in the surfactant solution.

The hydrodynamic size distribution of SiCH in aqueous solution was measured by the dynamic light scattering (DLS) method and using a mastersizer (Malvern zetasizer nano ZS90, Malvern, United Kingdom). The measurements were performed at 25.0 °C± 0.1 °C. The concentration of the samples was fixed at double CMC (above CMC), and all solutions were filtered through 0.2 μm micropore membranes before test.

The electrical conductivity was measured using a P904 PH conductivity meter at 283.15 − 303.15 K. The instrument was calibrated with a standard KCl solution prior to use. The conductivity values were recorded for surfactant solutions with concentrations ranging from 2 to 20 mmol/L to determine the critical micelle concentration (CMC).

Based on the Gibbs adsorption isotherm, the surface adsorption capacity was quantified, and the critical micelle concentration (CMC) was determined. The key interfacial parameters—including CMC, surface adsorption efficiency (PC_20_), and the area occupied per surfactant molecule (*A*_S_) at the air/water interface were tested and analyzed. Temperature-dependent measurements were performed to obtain the thermodynamic parameters of micellization ( ${\Delta G}_{m}^{0}$ , ${\Delta H}_{m}^{0}$ , $\Delta S_{m}^{0}$ ).

All experiments and measurements were performed as the mean of three measurements, and we have calculated the relative uncertainty (*u*_r_, %) of the tabulated values.

## 3 Results and discussion

### 3.1 Characterization of surfactant

**FIGURE 3:**
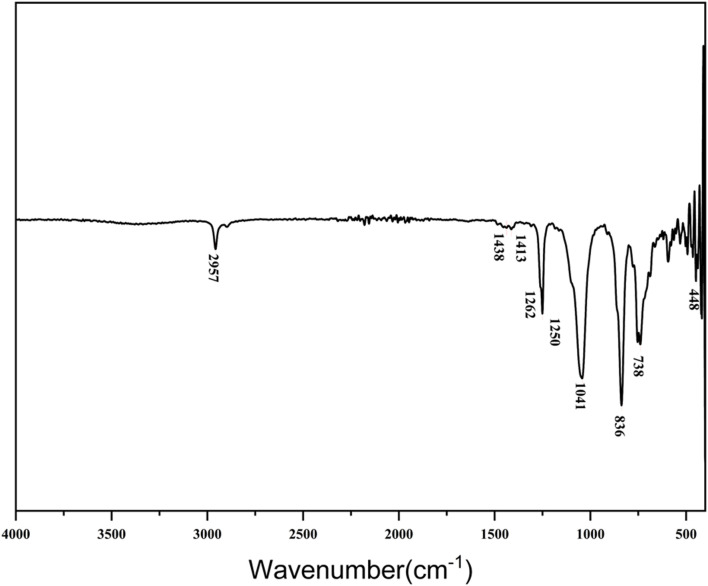
IR spectrum of SiCH.

The FT-IR spectrum of the synthesized organosilicon surfactant (Figure 3) exhibited characteristic absorption bands corresponding to key functional groups. The bands observed at 2,957 cm^-1^ and 1,413 cm^-1^ are assigned to the asymmetric stretching vibration (ν_as_ (CH_2_)) and the in-plane bending vibration (δ(CH_2_)) of aliphatic C–H bonds, respectively. Peaks at 1,485 cm^-1^ and 1,262 cm^-1^ correspond to the symmetric stretching vibration (ν_s_ (C–N)) of the C–N bond and the methyl rocking vibration (ρ(CH_3_)) of the quaternary ammonium group N^+^(CH_3_)_2_. Additionally, absorptions at 1,250 cm^-1^ and 738 cm^-1^ are attributed to the C–O stretching vibration (ν(C–O)) and the out-of-plane bending vibration (γ(O–H)) of the secondary alcohol group (–CH(R′)–OH).

Notably, intense absorption bands were observed at 1,041 cm^-1^ and 448 cm^-1^, corresponding to the asymmetric stretching vibration (ν_as_ (Si–O–Si)) and the bending vibration (δ(Si–O)) of the siloxane (Si–O–Si) linkage, respectively. A distinct peak at 836 cm^-1^ was assigned to the Si–C stretching vibration (ν(Si–C)).

**FIGURE 4:**
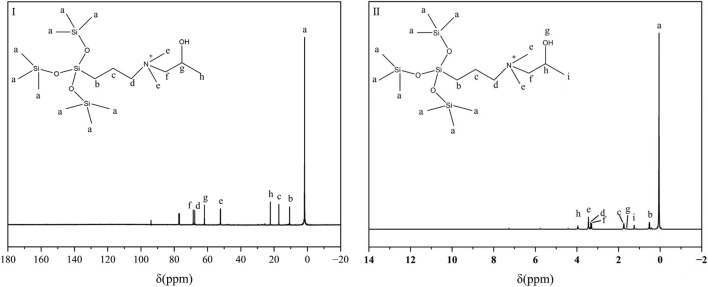
^13^C NMR(I) and ^1^H NMR(II) spectrum of SiCH.

**FIGURE 5:**
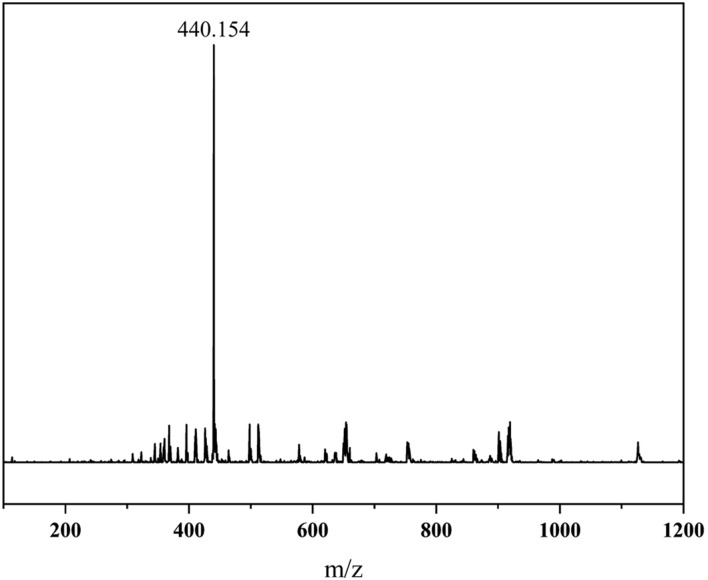
MS spectrum of SiCH.

Nuclear Magnetic Resonance (NMR) spectroscopy and Mass Spectrometry (MS) were employed to confirm the molecular structure of the synthesized organosilicon surfactant. The ^13^C NMR spectrum (Figure 4) exhibited 8 distinct carbon signals (a–h), consistent with the expected carbon framework. The ^1^H NMR spectrum displayed 9 well-resolved proton signals (a–i), with chemical shifts corresponding to the proposed structure. MS analysis in positive electrospray ionization mode (ESI^+^) showed an isotope-resolved molecular ion cluster centered at m/z 440.154, in close agreement with the theoretical monoisotopic mass for C_17_H_46_NO_4_Si_4_ [M]^+^. In Figure 5, the prominent [M]^+^ peak further corroborated the cationic nature of the quaternary ammonium moiety. Together with the FT-IR spectral data (Figure 3), these results provide conclusive evidence for the successful synthesis and structural integrity of the target organosilicon surfactant.

^1^H NMR (CDCl_3_, ppm): *δ* 3.95 (1H, N^+^CH_2_C*H*(OH)CH_3_), 3.44 (6H, N^+^(C*H*_*3*_)_*2*_), 3.33 (2H, SiCH_2_CH_2_C*H*_*2*_), 3.29 (2H, N^+^C*H*_*2*_CH(OH)CH_3_), 1.74 (2H, SiCH_2_C*H*_*2*_CH_2_), 1.59 (1H, N^+^CH_2_CH(O*H*)CH_3_), 1.25 (3H, N^+^CH_2_CH(OH)C*H*_*3*_), 0.51 (2H, SiC*H*_*2*_CH_2_CH_2_), 0.05 (27H, Si(C*H*_*3*_)_*9*_). ^13^C NMR (CDCl_3_, ppm): *δ* 68.62 (N^+^ *C*H_2_CH(OH)CH_3_), 67.70 (SiCH_2_CH_2_ *C*H_2_), 61.84 (N^+^CH_2_ *C*H(OH)CH_3_), 52.18 (N^+^(*C*H_3_)_2_), 22.25 (N^+^CH_2_CH(OH)*C*H_3_), 17.14 (SiCH_2_ *C*H_2_CH_2_), 10.65 (Si*C*H_2_CH_2_CH_2_), 1.65 (Si(*C*H_3_)_9_). MS-ESI (m/z): calc. 440.25; found 440.15.

### 3.2 Thermal stability of surfactant

**FIGURE 6:**
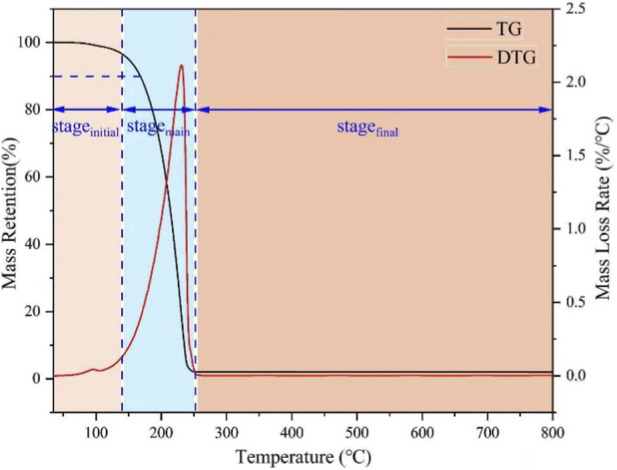
TG and DTG curves of SiCH.

The thermal degradation behavior of the organosilicon surfactant was evaluated by thermogravimetric analysis (TG) and derivative thermogravimetry (DTG), as illustrated in Figure 6. Based on the thermal data summarized in Table 1, the pyrolysis process can be categorized into three distinct stages: an initial decomposition phase, a main mass-loss phase, and a final residual decomposition phase.

**TABLE 1:** Thermal data of SiCH.

T_10%_ (°C)	T_max_ (°C)	PMLR (%/°C)	Mass loss (%)			Residue (%)
			Stage_initial_	Stage_main_	Stage_final_	
167.19	231.52	2.12	3.29	94.09	0.61	2.01

*u*_r(T)_ < 0.42%, *u*_r(Residue)_ < 1.73%.

#### 3.2.1 Initial stage (30 °C–140 °C)

A minor mass loss was observed, attributable primarily to the evaporation of adsorbed water and the onset of pyrolysis. The temperature corresponding to 10% mass loss (T_10%_), often regarded as the initial decomposition temperature, was determined to be 167 °C.

#### 3.2.2 Main stage (140 °C–255 °C)

This stage represents the principal pyrolysis region, characterized by a substantial mass loss of up to 94.09%. The DTG curve exhibits a pronounced peak mass loss rate (PMLR) of 2.12%/°C at 231.52 °C, accompanied by a noticeable endothermic effect, indicating rapid decomposition of the surfactant molecules.

#### 3.2.3 Final stage (255 °C–600 °C)

Pyrolysis was largely complete within this stage, with the residual mass stabilizing at 0.61%, suggesting near-complete volatilization of organic components.

Overall, SiCH demonstrates the well-defined PMLR peak, and low residual mass. The endothermic nature of the pyrolysis process may help retard thermal degradation, thereby preserving the active surfactant structure longer under high-temperature conditions, which is advantageous in fire-suppression applications. Moreover, Etz et al. investigated the high-temperature decomposition reactions and computed the bond dissociation enthalpies (BDEs) of trimethylsiloxane surfactants ([(CH_3_)_3_SiO]_2_Si(CH_3_) (CH_2_)_3_O(CH_2_)_2_OH) (Etz et al., 2022). From The BDEs, the high-temperature decomposition will initiate along the polyethylene glycol chain (82.3–94.7 kcal/mol), and leads to formation of stable polydimethylsiloxane-like products (the 89.6–139.6 kcal/mol) and small organics such as ethylene, formaldehyde, and acetaldehyde, etc. These decomposition products have a much smaller impact on the environment compared with fuorine-containing surfactants (Ahmed et al., 2020; Stoiber et al., 2020; Xiao et al., 2021). In this work, the surfactants also possess trimethylsiloxane and alkyl alcohol structures, which high-temperature decomposition products are similar to those reported in the literature. The results of TG and DTG indicate that the surfactant undergoes near-complete decomposition at high temperatures, leaving a minimal char residue of only 0.61%. This high volatility at pyrolysis temperatures suggests a lower potential for forming persistent, carbonized environmental contaminants compared to some polymeric materials, further supporting its profile as an environmentally favorable alternative to fluorocarbon surfactants.

### 3.3 Surface activity of surfactant

**FIGURE 7:**
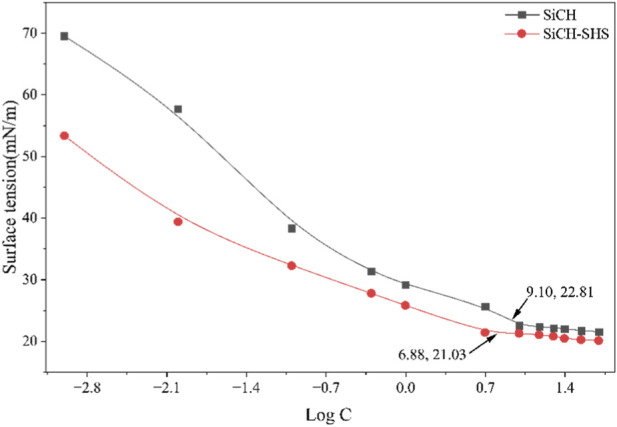
Plot of surface tension vs. log C for the pure SiCH aqueous system and SiCH-SHS mixed systems at 25 °C (molar ratio = 1:1)

Cationic organosilicon surfactants feature a distinct amphiphilic structure that drives their adsorption at the air/water interface in aqueous solution, primarily due to the hydrophobic effect (Tan et al., 2013a). This interfacial adsorption leads to a reduction in surface tension, with molecules forming a monolayer in which the hydrophobic groups extend into the air and the hydrophilic headgroups remain solvated in the aqueous phase (Tan et al., 2013b). The surface tension curves of SiCH at different concentrations are measured at 25 °C, and the results are shown in Figure 7. As illustrated in Figure 7, the surface tension of SiCH decreases with increasing concentration until reaching an inflection point, beyond which it stabilizes (Czajka et al., 2015). The corresponding critical micelle concentration (CMC) was determined to be 9.10 mmol/L, with the surface tension at CMC (γ_CMC_) of 22.81 mN/m. At low concentrations, surfactant molecules preferentially adsorb at the air/water interface. As concentration rises, interfacial packing becomes more compact, leading to a continuous decline in surface tension. When the surfactant concentration reaches the CMC, the air/water interface becomes saturated and can accommodate no additional molecules. Further increases in surfactant concentration result in the dissolution of excess molecules in the bulk solution, where they self-assemble into ordered aggregates. In these aggregates, the hydrophilic headgroups face the aqueous phase while the hydrophobic tails are directed inward, minimizing the system’s free energy (Huang et al., 2019).

The favorable intermolecular interactions in mixed surfactant system have motivated extensive research into their properties (Zhang et al., 2024). Studies on equimolar binary mixtures containing Gemini and conventional surfactants have demonstrated pronounced synergistic effects, particularly between quaternary ammonium cationic and anionic surfactants, which arise from electrostatic attraction between oppositely charged headgroups (Wang et al., 2024). The incorporation of hydrocarbon surfactants into organosilicon surfactant systems not only enhances surface activity through synergy but also reduces the required dosage of the latter, offering both economic and environmental benefits. The binary surfactant system was prepared by mixing SiCH and SHS at a molar ratio of 1:1 (equimolar) prior to dilution. As shown in Figure 7, the addition of sodium hexanesulfonate decreased the CMC and *γ*_CMC_ of the SiCH-SHS to 6.88 mmol/L and 21.03 mN/m respectively, confirming a strong synergistic interaction with the organosilicon surfactant. The combination of the organosilicon surfactant with sodium hexanesulfonate demonstrates a characteristic synergy typical of cationic/anionic surfactant systems (Mesa and Risuleo, 2021). Through synergistic interfacial adsorption and mixed micelle formation, the SiCH-SHS achieves a more compact and ordered molecular arrangement at air/water interface, leading to a significant reduction in surface tension and improved wetting and penetration performance (Wang et al., 2024). The flexible siloxane backbone of the organosilicon surfactant, which exhibits very low surface energy, facilitates rapid orientation and the formation of a dense interfacial film. Meanwhile, the short-chain sodium hexanesulfonate molecules fill the intermolecular voids within the adsorbed layer, enhancing its packing density. Additional electrostatic and hydrophobic interactions between the two components promote the formation of thermodynamically stable mixed micelles, thereby lowering the critical micelle concentration (CMC) and increasing interfacial activity. Moreover, the synergistic effect between two-component mixed micelles can be evaluated by the interaction parameter (*β* ^m^) using Rubingh’s equation (Rubingh, 1979).

$$\frac{{\left(x_{1}^{m}\right)}^{2}\ln\left(CMC_{12}\alpha_{1}/CMC_{1}x_{1}^{m}\right)}{{\left(1-x_{1}^{m}\right)}^{2}\ln\left[CMC_{12}\left(1-\alpha_{1}\right)/CMC_{2}\left(1-x_{1}^{m}\right)\right]}=1$$ (1)

$$\beta^{m}=\frac{\ln\left(CMC_{12}\alpha_{1}/CMC_{1}x_{1}^{m}\right)}{{\left(1-x_{1}^{m}\right)}^{2}}=\frac{\ln\left(CMC_{12}\alpha_{2}/CMC_{2}x_{2}^{m}\right)}{{\left(1-x_{2}^{m}\right)}^{2}}$$ (2)

where $x_{1}^{m}$ and $x_{2}^{m}$ are the micellar mole fractions of SiCH and SHS in the mixed micelles, respectively. CMC_1_, CMC_2_ and CMC_12_ are the critical micelle concentrations of SiCH, SHS and mixed systems, respectively. The values of CMC_1_, CMC_2_ and CMC_12_ can be measured are 9.10, 117.60 and 6.88 mmol/L, respectively. *α*_1_ (0.5) is the mole fraction of SiCH in the mixed system. $x_{1}^{m}$ is calculated by the iterative method based on Equation 1, and the result is 0.672. Therefore, the *β* ^m^ = −5.35 is obtained based on Equation 2. The *β* ^m^ value is negative implying a good interaction between the two surfactants, and a synergistic effect exists during the formation of the micelles.

The adsorption behavior of surfactants at the liquid–gas interface can be described using the Gibbs adsorption equation. The saturated adsorption capacity (Γ_max_) and the minimum surface area per molecule (*A*_S_) were determined according to Equations 3, 4, respectively (Regev and Guillemet, 1999):

$$\Gamma_{\max}=-\frac{1}{2.303iRT}{\left(\frac{d_{\gamma }}{d_{\log C}}\right)}_{T,P}$$ (3)

$$A_{S}=\frac{1}{N_{A}\Gamma_{\max}}$$ (4)

Where *N*_*A*_ represents the Avogadro constant (6.02 × 10^23^ mol^-1^), *R* is the universal gas constant (8.31 J/(mol·K)), and *T* denotes the absolute temperature (K). The term *γ* corresponds to the surface tension (mN/m), and *C* is the surfactant concentration (mmol/L). The value of d*γ*/dlog*C* corresponds to the slope of the *γ*-log*C* curve. A negative slope (d*γ*/dlog*C*<0) yields a positive surface excess concentration (Γ_max_ > 0), confirming the accumulation of surfactant molecules at the interface. For univalent ionic surfactant, when the concentrations of cations and anions in solution are equal, the prefactor ‘i’ is taken as 2. Generally, a larger value of Γ_max_ corresponds to a smaller value of *A*_S_ (the area occupied per molecule), which signifies a more compact arrangement of surfactant molecules at the liquid-air interface (Wang et al., 2017; Zhu et al., 2020). The maximum surface excess concentration and the average area per molecule at the interface for these surfactant systems at 25 °C are presented in Table 2.

**TABLE 2:** The surface activity parameters of surfactants at 25 °C.

Surfactants	CMC (mmol/L)	*γ*_CMC_ (mN/m)	d*γ*/dlg*C*	Γ_max_ (μmol/m^2^)	*A*_S_ (nm^2^)	PC_20_
SiCH	9.10	22.81	−12.75	1.12	1.48	1.80
SiCH-SHS	6.88	21.03	−15.29	1.34	1.24	2.91

*u*_r(CMC)_ < 0.74%, *u*_r(*γ*CMC)_<1.58%.

In terms of critical micelle concentration (CMC), the SiCH-SHS exhibits the lowest value (6.88 mmol/L), which is substantially lower than those of the individual organosilicon surfactant (9.10 mmol/L). This result demonstrates a strong synergistic interaction between SiCH and SHS, promoting more efficient micellization that can be attributed to electrostatic screening between polar headgroups and enhanced hydrophobic interactions. In terms of surface tension reduction efficiency, as reflected by the *γ*_CMC_ values, SiCH showed a higher *γ*_CMC_ of 22.81 mN/m and a more expanded interfacial arrangement (*A*_S_ = 1.48 nm^2^). The SiCH-SHS attained an intermediate *γ*_CMC_ of 21.03 mN/m, which is notably lower than that of SiCH alone. The corresponding reduction in *A*_S_ to 1.24 nm^2^ further indicates enhanced molecular packing density at the interface due to the synergistic effect of compounding. In order to evaluate the differences in surface activity between the organosilicon surfactants used in this work and the fluorocarbon and hydrocarbon surfactants, we have collected relevant literature data on various quaternary ammonium salt surfactants and listed them in Table 3. As shown in Table 3, the *γ*_CMC_ values of fluorocarbon and organosilicon surfactants is significantly higher than that of hydrocarbon surfactants. The *γ*_CMC_ values of fluorocarbon surfactants can be as low as below 20 mN/m (16.91–18.49 mN/m), and organosilicon surfactants are second only to fluorocarbon surfactants (mostly ranging from 20 to 30 mN/m). It can be seen that the surface tension of the surfactant in this work (22.81 mN/m) is comparable to that of fluorocarbon surfactant, and it exhibits excellent surface properties. Furthermore, the *A*_S_ value (1.48 nm^2^) in this work is slightly higher than most of most other surfactants in Table 3 (0.49–1.75 nm^2^). The large *A*_S_ value indicates that the arrangement of surfactant molecules at the air/water interface is relatively loose, which probably are related to the so-called “umbrella” conformation formed by the bulky trimethylsilyoxyl groups on the water surface, resulting in a greater distance between the surfactant molecules (Schmaucks et al., 1992; Snow et al., 1991).

**TABLE 3:** Comparison of CMC, γCMC, Γ_max_ and AS parameters in aqueous solution at 25 °C.

Surfactants	CMC (mmol/L)	*γ*_CMC_ (mN/m)	Γ_max_ (μmol/m^2^)	*A*_S_ (nm^2^)	Reference
Si_3_PyCl	23.90	24.50	2.48	0.67	Tan et al. (2018)
Si_4_pyrCl	6.50	20.30	1.41	1.18	Fang et al. (2016)
Si_n_N_2_Cl_2_	0.61–9.74	29.03–29.74	1.70–2.33	0.71–0.97	Hao et al. (2015)
Vi-Si_3_PyCl	13.50	22.30	2.05	0.81	Tan et al. (2019)
Vi-Si_4_PyrCl	1.60	21.70	1.08	1.51	Tan et al. (2019)
SFHCS	2.15	18.49	1.24	1.34	Zhu et al. (2020)
PFPE-B	0.07	16.87	2.16	0.77	Shen et al. (2018)
FC-4	23.50	16.91	1.23	1.35	Zhu et al. (2024)
C_14_TAB	3.00	38.00	3.40	0.49	Simister et al. (1992)
C12Bn	0.26	29.23	0.95	1.75	Shaban et al. (2016)
C10mimBr	29.30	39.70	1.72	0.97	Dong et al. (2007)
SiCH	9.10	22.81	1.12	1.48	This work

The efficiency of reducing surfactant tension can be evaluated using the pC_20_ value, which is defined as the negative logarithm of the surfactant concentration required to reduce the surface tension of the solvent by 20 mN/m. Reveals that the SiCH-SHS attains a markedly high value of 2.91, which is significantly higher than those of SiCH surfactant (1.80). This indicates that the SiCH-SHS effectively lowers the surface tension of water even at very low concentrations, exhibiting superior adsorption efficiency and further corroborating the synergistic interaction between its components.

### 3.4 Aggregation behavior of surfactant in solution

**FIGURE 8:**
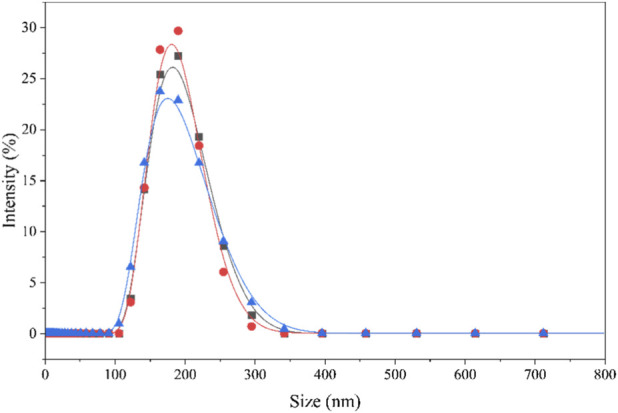
Intensity-percent size distribution of SiCH with double CMC at 25 °C.

To gain a deeper understanding of the self-assembly behavior of surfactants in aqueous solutions, we used dynamic light scattering (DLS) to characterize the size distribution of their aggregates. DLS is an effective method for accurately measuring the hydrodynamic size of nanoparticles by analyzing the fluctuations in the light scattering signal caused by the Brownian motion of particles in solution (Zhao et al., 2023). This study, using DLS analysis, aimed to determine the average size, size uniformity, and stability of micelles formed by SiCH at specific concentrations. The detailed measurement results and analysis are shown in Figure 8.

Figure 8 shows the intensity percentage particle size distribution from three independent measurements, which shows a typical unimodal distribution, and the size distribution of SiCH is between 122 and 295 nm. The size distribution of is much larger than that of spherical micelles (Shen et al., 2018), indicating the non-uniform sized spheroidal aggregates have formed in solution. The results from the three replicate experiments were highly consistent, with the particle size distribution peak centered at approximately 185 nm and the primary distribution range being between 122 and 295 nm. The polydispersity index (PDI) is 0.193, which is slightly higher than the reported PDI values of organosilicon surfactants (0.096 − 0.164) (Meng, 2023). The PDI value is more than 0, confirming that the aggregates formed by the surfactant in the solution are non-uniform. (Meng et al., 2022). This result demonstrates that this surfactant can stably self-assemble into ordered aggregates of approximately 185 nm under the experimental conditions. Theoretically, the structures of surfactant aggregates in solution can be estimated by the critical “packing parameter” (Regev and Guillemet, 1999):

$$P=v/a_{0}l_{c}$$ (5)

**FIGURE 9:**
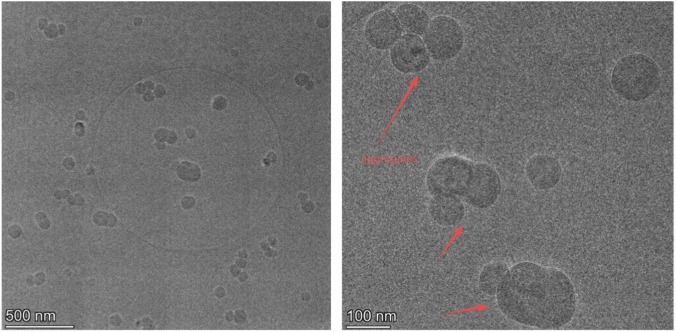
Cryo-TEM images of aggregates of SiCH.

Where *l*_c_ (nm) is the critical chain length of the surfactant, *v* (nm^3^) is the volume of the tail group of the surfactant, and *a*_0_ (nm^2^) is the area of per surfactant head group (here, the value of *a*_0_ can be instead by *A*_S_ of 1.48 nm^2^). As a rough estimate, *l*_c_ and *v* can be calculated using the formulas *l*_c_ = 0.154 + 0.1265*n*, and *v* = 0.0274 + 0.0269*n*, where *n* is the number of atoms in the hydrophobic chain. The calculations of the values for organosilicon surfactant give *l*_c_ = 1.04 nm and *v* = 0.22 nm^3^. Then, *P* = 0.14 can be obtained based on Equation 5. In general, when *P* < 1/3, 1/3 < *P* < 1/2, and 1/2 < *P* < 1, the surfactant molecules are inclined to form spherical, rod-like, and vesicles respectively. The *P* value of SiCH is relatively small, indicating that the surfactant molecules in the aqueous solution are more prone to self-assemble into spherical aggregates. Moreover, the micromorphology of the surfactant micelles in aqueous solution are observed in Figure 9. We can clearly observe that the surfactant can self-assemble into non-uniform sized spheroidal aggregates, and the size of these aggregates are consistent with the results of DLS analysis.

### 3.5 Thermodynamic analysis of surfactant micelles

**FIGURE 10:**
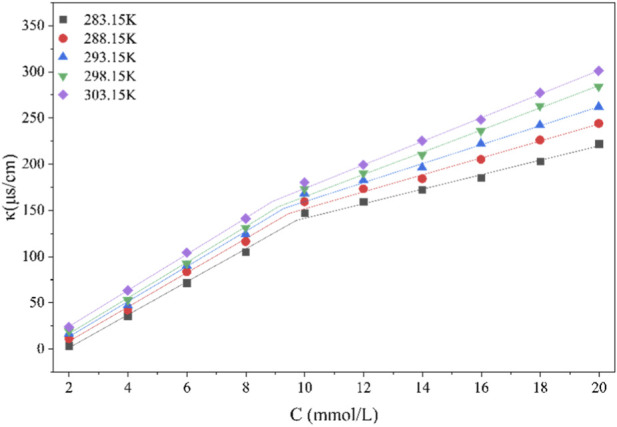
Plots of specific conductivity (*κ*) against the concentration of SiCH.

While surface tension measurements at a fixed temperature offer valuable information on interfacial activity, a systematic thermodynamic analysis of micellization across a range of temperatures is essential to elucidate the fundamental driving forces—such as enthalpy or entropy contributions—governing self-assembly. It is critical for predicting surfactant performance stability under varying environmental conditions. Conductivity measurement represents a precise and reliable method for determining the critical micelle concentration (CMC) of ionic surfactants at different temperatures, as it is highly sensitive to changes in the concentration and mobility of charge carriers in solution. The critical micelle concentration (CMC) and related parameters were determined by measuring the specific conductivity (*κ*) of aqueous organosilicon surfactant solutions across a range of molar concentrations (C) at five temperatures (283.15, 288.15, 293.15, 298.15, and 303.15 K). The relationship between *κ* and C at these temperatures is presented in Figure 10.

Figure 10 clearly displays two well-defined linear segments in the conductivity curve at each temperature. At low concentrations (below the CMC), surfactant molecules exist predominantly as monomers. Consequently, the solution behaves as a strong electrolyte, with the specific conductivity (κ) increasing linearly with concentration. Upon exceeding this threshold concentration, surfactant molecules undergo self-assembly into micelles, marking the transition to the high-concentration regime.

Micellar solutions represent thermodynamic equilibrium systems amenable to analysis by established thermodynamic frameworks. Two commonly employed models are the mass action model and the phase separation model. The mass action model, which explicitly accounts for counterion binding to the micellar surface, is particularly suitable for describing ionic surfactant systems. The standard Gibbs free energy of micellization ( ${\Delta G}_{m}^{0}$ ), the standard enthalpy of micellization ( ${\Delta H}_{m}^{0}$ ), and the standard entropy of micellization ( $\Delta S_{m}^{0}$ ) can be calculated according to Equations 6–8 respectively (Ju et al., 2017; Sastry et al., 2012; Shen et al., 2018):

$${\Delta G}_{m}^{0}=\left(1+\beta \right)RTln\chi_{CMC}$$ (6)

$${\Delta H}_{m}^{0}=-\left(1+\beta \right)RT^{2}\frac{dln\chi_{CMC}}{dT}$$ (7)

$$S_{m}^{0}=\frac{{\Delta H}_{m}^{0}-{\Delta G}_{m}^{0}}{T}$$ (8)

Where χ_CMC_ is the critical micelle concentration in mole fraction units can be calculated using χ_CMC_ = CMC/(moles of water + moles of SiCH), *β* represents the degree of counterion binding, *β* is correspondingly defined as *β* = 1–*α*, quantifying the proportion of counterions electrostatically bound to the micelle surface. The value of *β* is critically important, as it reflects the extent of charge neutralization at the micellar interface. The micelle dissociation degree (*α*) was determined from the ratio of the slopes obtained by linear regression analysis of the conductivity curves before (slope = S_1_) and after (slope = S_2_) the critical micelle concentration, such that *α* = S_2_/S_1_. This parameter represents the fraction of counterions that remain free in solution following micellization. The *β* values of organosilicon surfactants with different structures are different. The *β* values of 0.55 − 0.69 were reported for organosilicon bi-quaternary ammonium surfactants (Hao et al., 2015). While the *β* values of the trisiloxane ionic surfactants is 0.26 − 0.39 (Tan et al., 2019). The *β* values (0.35 − 0.56) of SiCH are all relatively low, indicating that the self-repulsion between the head groups and counterions is more significant than the attraction. Moreover, the β decreases slightly with the temperature increases. This is mainly caused by the thermal motion of the surfactant molecules, which is consistent with the alkane-based cationic surfactants (González-Pérez et al., 2004; Zhang et al., 2012).

Table 4 details the various micellization parameters measured by conductivity at different temperatures. It shows that the micellization process of SiCH is a spontaneous, entropy-driven thermodynamic behavior. Experimental data show that the standard Gibbs free energy of micellization ( ${\Delta G}_{m}^{0}$ ) remains consistently negative within the temperature range of 283.15–303.15 K, confirming the spontaneity of the process. The trends of ${\Delta G}_{m}^{0}$ (−29.63 − -31.78 kJ/mol), ${\Delta H}_{m}^{0}$ (4.21 − 4.32 kJ/mol) and $-T\Delta S_{m}^{0}$ (−33.89 − -36.09 kJ/mol) from Table 4 are consistent with the literature reported ( ${\Delta G}_{m}^{0}$ of −22.10 − -23.79 kJ/mol, ${\Delta H}_{m}^{0}$ of 0.60 − 28.07 kJ/mol and $-T\Delta S_{m}^{0}$ of −24.39 − -50.16 kJ/mol) (Tan et al., 2018). Although the enthalpy change ( ${\Delta H}_{m}^{0}$ ) of micellization is positive, indicating an endothermic process, which is energetically unfavorable, the entropic contribution ( $-T\Delta S_{m}^{0}$ ) generated by the large positive entropy change ( $\Delta S_{m}^{0}$ ) is sufficient to offset the unfavorable enthalpy change, becoming the primary driving force for the overall self-assembly process (Che et al., 2015; Tan et al., 2018). The values of ${\Delta H}_{m}^{0}$ are positive, suggesting the water structured around the trimethylsilyl hydrophobic group of trimethylsiloxane surfactants was destroyed. This increased the disorder of the organosilicon surfactants–solution system, resulting in a positive contribution to the values of $\Delta S_{m}^{0}$ . This phenomenon is a typical manifestation of the hydrophobic effect, whereby the hydrophobic tails of the surfactant aggregate in the aqueous phase, releasing the highly ordered water molecules previously bound around the tails, significantly increasing the overall disorder of the system. Furthermore, Meng et al., (Meng et al., 2023), measured that the ${\Delta G}_{m}^{0}$ value of anionic trimethylsiloxane surfactants was negative (−23.03 − -35.40 kJ/mol), and $-T\Delta S_{m}^{0}$ is also the primary driving force for the overall self-assembly process. However, the increase in temperature causes the CMC of the surfactants to rise slightly, which is inconsistent with the variation of CMC of cationic surfactants in this work with temperature. The reason is that the increase in temperature weakens the hydration layer of the cationic organosilicon surfactants head group, facilitating the formation of aggregates, and this effect predominates.

**TABLE 4:** Values of ${\Delta G}_{m}^{0}$ , ${\Delta H}_{m}^{0}$ , $\Delta S_{m}^{0}$ and $T\Delta S_{m}^{0}$ for the studied SiCH/SiCH-SHS in aqueous system at various temperatures.

T(K)	CMC (mmol/L)	β	${\Delta G}_{m}^{0}$ (kJ/mol)	${\Delta H}_{m}^{0}$ (kJ/mol)	$-T\Delta S_{m}^{0}$ (kJ/mol)	$\Delta S_{m}^{0}$ (J/mol/K)
283.15	9.73	0.56	−31.78	4.31	−36.09	127.46
288.15	9.45	0.50	−31.27	4.30	−35.57	123.44
293.15	9.34	0.46	−30.90	4.32	−35.21	120.11
298.15	9.10	0.38	−29.73	4.21	−33.95	113.87
303.15	8.91	0.35	−29.63	4.26	−33.89	111.79

*u*_r(CMC)_ < 0.63%, *u*_r_( ${\Delta G}_{m}^{0}$ )<0.50%, *u*_r_( ${\Delta H}_{m}^{0}$ )<1.21%, *u*_r_( ${\Delta S}_{m}^{0}$ )<0.73%.

## 4 Conclusion

The synthesized surfactant with a hydroxyl group demonstrated an initial decomposition temperature of 167 °C with a low residual mass, the high volatility at pyrolysis temperatures suggests a lower potential for creating persistent environmental contaminants. The surfactant effectively reduces surface tension with a CMC of 9.10 mmol/L. Through synergistic interfacial adsorption and mixed micelle formation, the SiCH-SHS mixed system achieves a more compact and ordered molecular arrangement at air/water interface, increasing the efficiency of reducing surfactant tension (PC_20_ = 2.91). DLS and Cryo-TEM analysis showed that the surfactant can self-assemble into non-uniform sized spheroidal aggregates, and the size distribution is between 122 and 295 nm (PDI = 0.193). Moreover, Thermodynamic parameters of ${\Delta G}_{m}^{0}$ < 0, ${\Delta H}_{m}^{0}$ > 0 and ${\Delta S}_{m}^{0}$ > 0, indicating that the micellization process is a spontaneous, entropy-driven process, characteristic of the hydrophobic effect.

## Data Availability

The original contributions presented in the study are included in the article/supplementary material, further inquiries can be directed to the corresponding author.
